# Epidemiological factors for type 2 diabetes mellitus: evidence from the Global Burden of Disease

**DOI:** 10.1186/s13690-021-00632-1

**Published:** 2021-06-22

**Authors:** Artur Kotwas, Beata Karakiewicz, Paulina Zabielska, Sylwia Wieder-Huszla, Anna Jurczak

**Affiliations:** 1 Subdepartment of Social Medicine and Public Health, Department of Social Medicine, Pomeranian Medical University in Szczecin, Szczecin, Poland; 2 Department of Clinical Nursing, Pomeranian Medical University in Szczecin, Szczecin, Poland

**Keywords:** diabetes mellitus, YLL, YLD, DALY

## Abstract

**Background:**

The United Nations acknowledged diabetes as an epidemic of the 21st century. Global trends demonstrate a continuing growth in its prevalence at approximately 2.5 % per year. The aim of the study was to analyse selected epidemiological factors for type 2 diabetes mellitus in Poland, Central Europe and the World.

**Methods:**

This study presents Global Burden of Disease (GBD) data. Study describes the type 2 diabetes burden in the studied populations based on years lived with disability (YLD), years of life lost (YLL), and disability-adjusted life years (DALYs).

**Results:**

Type 2 diabetes has been demonstrated to be a determinant of reduced life expectancy, as in the analysed period the condition presented an increasing trend, compared to other diseases.

**Conclusions:**

In recent years the observed YLL, YLD and DALY values for type 2 diabetes have been comparable to the expected ones. Thus the prognosis presented by GBD may be used as a reliable source of information and a basis for a health policy that reduces the number of patients with diabetes and related complications, comorbidities or mortality.

## Background

The *World Health Organization* reports that some 415 million people worldwide live with diabetes [1]. Global trends suggest a steady rise of the prevalence rate by about 2.5 % a year. According to estimates, the number of people with diabetes in Poland is in the range of 2.5–3.5 million. The reports submitted to the National Health Fund (*Narodowy Fundusz Zdrowia*, NFZ) do not paint a comprehensive picture of diabetes epidemiology. They provide for diagnosed cases, failing to account for patients without a confirmed diagnosis. It is estimated that undiagnosed diabetes may affect about 1 million people in Poland. It usually takes 5–6 years before the condition is diagnosed. Moreover, based on research findings authors report that approx. 5 million people are at risk for developing diabetes [1–3]. Some 20 years ago, diabetes was recognised as a 21st-century epidemic by the *United Nations* (UN) [2].

*Diabetes mellitus* (DM) is defined as a chronic metabolic disease, characterised by high blood glucose levels resulting from defects in insulin secretion and/or action [4]. Based onetiology and pathophysiology, two broad forms of diabetes are identified: type 1 diabetes and type 2 diabetes, with the latter accounting for about 90 % of all the cases. In this paper, we have decided to focus on type 2 diabetes. It develops in adults, notably older adults, for many years, often unnoticed and undiagnosed. The condition can and should be treated by diet modification and increased physical activity. At an advanced stage, the disease requires pharmacological treatment with blood glucose-lowering medications or insulin therapy. Diagnosis is often incidental or due to the onset of long-term diabetic complications in the form of coronary atherosclerotic plaque.

The risk of developing type 2 diabetes is increased by overweight/obesity (60 % of people with diabetes are overweight and 20 % are obese), unhealthy eating habits, low levels of physical activity [5–7]. According to epidemiological data, diabetes causes an average reduction of lifespan by 6 years, and the life expectancy of a 60-year-old individual affected by diabetes and cardiovascular disease is 12 years shorter than that in the general population [5, 8, 9]. Along the years of its course, the disease leads to long-term disability and significant impairment in quality of life. Moreover, one of the essential determinants to be considered is the risk of a considerable shortening of life expectancy [4, 10]. With a steadily growing number of patients and a high risk of dangerous complications, diabetes is classified as a lifestyle disease. It is one of the most common causes of death in highly developed countries and has been recognised as one of the top public health priorities. The unique characteristics of diabetes call for an interdisciplinary approach [6]. The study wasundertaken to analyse selected epidemiological indicators (Years of Life Lost – YLL, Years Lived with Disability – YLD, Disability-Adjusted Life Years – DALY) for type 2 diabetes in Poland, Central Europe and the World.

## Methods

Among many databases kept by national and supranational institutions, one of the most important and most advanced resources is the *Global Burden of Disease* (GBD) led by the *Institute for Health Metrics and Evaluation*, University of Washington. The aim of the project is to collect data on an ongoing basis and provide important and comparable information on the health status of the global population. The information is based on health indicators, both standard and complex. The main standard indicators measure morbidity, mortality, life expectancy. Complex measures include Years Lived with Disability (YLD), Years of Life Lost (YLL), Disability-Adjusted Life Years (DALY). Data regarding the health status of the global population have been estimated and collected since 1990. The findings are updated annually to ensure accuracy. In 2019, the data on the burden of disease were captured for 204 countries, 369 diseases. The assessment includes prevalence of diseases, as well as their risk factors, allowing for comparison according to geographical location, time, by sex and age. In the present study, GBD data were taken for: 2000, 2005, 2010, 2015 and 2019 for Poland, Central Europe and the World. The design of the study provided for comparing selected epidemiological indicators for Poland, a Central European country, with the metrics for Central Europe and global figures. The rise of the disease in the 21st century determined the choice of the years included in the analysis in 5-year intervals, starting from 2000. The most recent data published by the GBD refer to the year 2019.

Detailed information about the sources of data, methodology of estimation and statistical analyses are described in articles dedicated to the GBD methodology [9–11]. The burden of type 2 diabetes in the studied populations was described based on the metrics of YLL, YLD and DALY. In order to make the data comparable, age-standardised rates are presented, based on the GBD standard. We believe such an approach is justified by the need to learn about the pace of change since the beginning of the 21st century in three identified reference groups. The choice of groups was motivated by the differentiation in terms of GDP per capita, public health expenditure and human development index (HDI). Details are provided at level 4 (on a scale from 1 to 4) for cases of type 2 diabetes, and level 3 for risk factors. The collected data include both expected and observed values. On their basis, the ratio of observed-to-expected cases of type 2 diabetes was calculated for the analysed geographical locations and time intervals. The GBD database does not provide for the typical confidence range, instead presenting the 95 % uncertainty interval [11]. The analyses did not require the approval of the Bioethics Committee, as they were based on existing data.

## Results

Diabetes mellitus, like other chronic conditions, may lead to a number of complications, including premature death, which occurs earlier than in people who do not have the disease and at an earlier-than-normal age. Hence, prevention measures should provide for monitoring changes in the health status of the population. The objectives of these public health measures are not only quantitative, aimed at extending the duration of life, but also qualitative, that is extending the number of years lived in good health, free from disability or limitations in carrying out the activities of daily living due to one’s health. Information on premature mortality is contained in one of the key instruments for measuring the burden of disease, namely the DALY indicator. The concept of the DALY was authored and developed by public health experts – Prof. Christopher Murray of Harvard University and Prof. Alan Lopez from the University of Melbourne. The DALY represents the number of years of healthy life, which have been lost due to living with a disease or disability of specific severity and duration (YLD –*Years Lived with Disability*) plus the number of years of life lost due to premature mortality (YLL – *Years of Life Lost*) [10, 11].

**Fig. 1:**
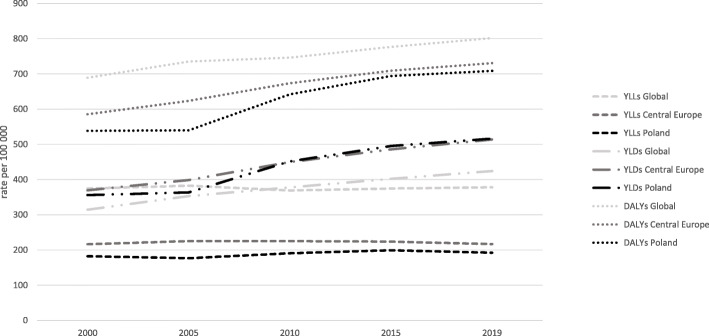
Increase of age-standardized rates (per 100,000) by location, for both sexes combined of YLLs, YLDs and DALYs for diabetes mellitus type 2 in Poland, Central Europe and Global

The data included in Table 1; Fig. 1 present the changes in YLL, YLD and DALY measures observed in 2000–2019 in Poland, Central Europe and Globally. The indicatorsare presented in figures per 100,000 people with the uncertainty interval, and the rates of change (presentation of trends) take the preceding period as the basis for comparison (chained indexing). The ranking for type 2 diabetes in terms of the analysed indicators is also shown. The number of years lost (YLL) in Poland in the year 2000 was less than half of the global metric, accounting for 48.60 % of the global figure – 182,00 YLL (95 % UI: 173.11-191.35) vs. 374.48 YLL (95 % UI: 356.63-389.94). The difference between Poland and the Central European average was much less pronounced (84.28 %). The YLL figures for the respective geographical regions were used to rank type 2 diabetes according to the impact of the disease on years of life lost. In 2000, the disease was ranked 24th in Poland, 22nd in Central Europe and 24th Globally. To put it in perspective, it was ranked higher than breast cancer or years of life lost to hypertension-mediated heart disease. Over the years, the YLLs increased in each area under analysis. In Poland, the largest upswing was observed in 2005–2010 (7.96 %). Over the course of the analysed period, type 2 diabetes came to weigh more and more heavily on the years of life lost: Globally up 9 spots, in Central Europe up 8, and in Poland – up 6 spots.

In terms of the number of years lived with disability (YLD), the metric for Poland in the year 2000 was similar to the other analysed regions – 355.96 YLD (95 % UI: 242.76-481.67). This accounted for 113.26 % of the Global YLD Fig. (314.28 YLD, 95 % UI: 217.26-423.18) and 96.33 % of that for Central European countries (369.49 YLD, 95 % UI: 247.30-510.58). Type 2 diabetes in Poland, same as in Central Europe as a whole, was the 5th leading cause impacting on the number of years lived with disability, and the 8th worldwide. Throughout the period under analysis, Poland saw dynamic changes in the pace of growth, ranging from 2.09 to 24.18 %. Global data are characterised by a decelerating growth rate, from 12.25 % to 2005 − 2000 to 6.55 % for 2019–2015. In 2019, compared to the baseline year 2000, similar differences were noted in YLD figures for the analysed regions. In Poland, the indicator reached the highest figure – 516.33, which accounted for 100.55 % of the value calculated for Central Europe and 121.85 % of the Global figure. In the ranking, type 2 diabetes in 2019 climbed 2 spots in the Global ranking (to rank 6), and in Poland and Central Europe is was one spot up, to rank 4.

The DALY metric – disability-adjusted life years, in 2000 was at its highest in the Global dimension (688.76 DALY, 95 % UI: 589.23-803.18), it was 15 % lower in Central European countries (585.45 DALY, 95 % UI: 464.14-726.28), and 21.89 % lower in Poland (537.96 DALY, 95 % UI: 426.00-666.14). The considerable difference between the Global, Central Europe and Poland figures is also seen in the ranking, respectively: rank 15, 9 and 11. In the period under analysis, the Global DALY rose by 16.37 %, the Central European metric by 24.73 %, and Poland by 32.65 %. In the Global ranking, type 2 diabetes jumped 7 places (up to rank 8), in Poland 5 places (up to rank 6), and 4 places in Central Europe (rank 5).

The selected metrics (YLL, YLD, DALY) were also examined in terms of observed-to-expected figures – Table 2. With regard to the years of life lost (YLL), in 2000 the observed figures were less than half of the expected, across the board in Poland, Central Europe and Globally. With the passage of time, the difference between projections and the actual figures diminished, especially in the case of Poland. Already by 2015, the observed-to-expected ratio for YLL in Poland amounted to 0.93, while the Global ratio remained much lower – 0.69. In 2019, the ratio for Poland held steady at 0.93, amounting to 0.85 for Central Europe, with 0.71 at the Global level. With regard to the years lived with disability (YLD), the differences between the analysed regions were more pronounced. At baseline,year2000, there were considerable differences in the observed-to-expected ratio, from 0.63 Globally to 0.81 for Central Europe, with Poland at 0.75. By the end of the observation period, the metric for Poland was much higher. The observed figures nearly matched the projections (0.99). In the case of Central Europe, the actual figures exceeded the expectations. Globally, in turn, the ratio went up to 0.79 in the period under analysis. Last but not least, the DALY figures were examined, too. At baseline, the observed figures were much lower than the expectations, ranging from 0.58 Globally to 0.71 for Central Europe. A gradual increase was observed in all the regions under analysis. In Poland, the ratio was up to 0.98 in 2019, which was the highest ratio in the analysed regions.

**Table 1:** Age-standardized YLL, YLD, and DALY rates for diabetes mellitus type 2 (level 4) for Poland, Central Europe and Global, for both sexes combined, in 2000, 2005, 2010, 2015 and 2019

Category		Age-standardized YLLs rate per 100,000			Age-standardized YLDs rate per 100,000			Age-standardized DALYs rate per 100,000		
year	Cause	**Global (GL)**	**Central Europe (CE)**	**Poland (PL)**	**Global (GL)**	**Central Europe (CE)**	**Poland (PL)**	**Global (GL)**	**Central Europe (CE)**	**Poland (PL)**
2000	Value	374.48	215.96	182.00	314.28	369.49	355.96	688.76	585.45	537.96
	upper/lower bounds	356.63-389.94	207.66-223.49	173.11-191.35	217.26-423.18	247.30-510.58	242.76-481.67	589.23-803.18	464.14-726.28	426.00-666.14
	Rank	24	22	24	8	5	5	15	9	11
**% change in age-standardized rates**		-	-	-	-	-	-	-	-	-
2005	Value	382.34	224.86	176.33	352.79	398.57	363.38	735.13	623.43	539.71
	upper/lower bounds	365.04-398.53	215.66–231.40	166.79-184.38	243.41-474.89	266.84-552.91	248.59-492.64	623.06-864.51	493.34-777.27	426.18-671.17
	Rank	20	19	23	8	4	5	13	7	9
**% change in age-standardized rates**		2.10 %	4.12	-3.12 %	12.25 %	7.87 %	2.09 %	6.73 %	6.49 %	0.33 %
2010	Value	368.85	224.86	190.36	377.15	449.66	451.24	746.00	673.44	641.60
	upper/lower bounds	352.33-383.96	215.66–231.40	178.96-198.09	260.70-507.64	302.64-617.42	311.34-608.37	629.18-878.33	528.15-841.08	501.62-800.61
	Rank	15	19	19	7	4	5	13	6	7
**% change in age-standardized rates**		-3.53	-0.48 %	7.96 %	6.91 %	12.82 %	24.18 %	1.48 %	8.02 %	18.88 %
2015	Value	374.47	223.78	199.08	401.86	485.59	494.86	776.33	709.06	693.94
	upper/lower bounds	356.43-390.15	213.31-230.12	186.50-206.90	276.61-541.79	326.86-670.64	339.66–672.90	650.37–918.00	550.56-892.62	539.93-873.66
	Rank	15	17	18	6	4	3	10	5	6
**% change in age-standardized rates**		1.52	-0.14 %	4.58 %	6.55 %	7.99 %	9.67 %	4.07 %	5.29 %	8.16 %
2019	Value	377.82	216.77	191.89	423.72	513.46	516.33	801.55	730.22	708.22
	upper/lower bounds	354.92-402.21	188.11-248.63	151.57–226.40	289.46-576.59	344.58-707.28	352.55-704.02	670.58-954.43	558.96-923.08	535.50-902.47
	Rank	15	14	18	6	4	4	8	5	6
**% change in age-standardized rates**		0.90	-3.00	-3.61 %	5.44 %	5.74 %	4.34 %	3.25 %	2.98 %	2.06 %

**Table 2:** YLLs, YLDs and DALYs for diabetes mellitus type 2 in Poland, Central Europe and Global with the ratio of observed to expected (OER) age-standardized rates by location, for both sexes combined

	YLLs			YLDs			DALYs		
	**Global (GL)**	**Central Europe (CE)**	**Poland (PL)**	**Global (GL)**	**Central Europe (CE)**	**Poland (PL)**	**Global (GL)**	**Central Europe (CE)**	**Poland (PL)**
2000	0.51^a^	0.49^a^	0.49^a^	0.63^b^	0.81^c^	0.75^c^	0.58^b^	0.71^c^	0.67^b^
2005	0.57^b^	0.61^b^	0.60^b^	0.70^c^	0.86^d^	0.82^c^	0.65^b^	0.79^c^	0.76^c^
2010	0.60^b^	0.74^c^	0.77^c^	0.74^c^	0.91^d^	0.84^c^	0.69^b^	0.87^d^	0.82^c^
2015	0.69^b^	0.86^d^	0.93^d^	0.78^c^	0.98^d^	0.93^d^	0.75^c^	0.95^d^	0.93^d^
2019	0.71^c^	0.85^d^	0.93^d^	0.79^c^	1.00^e^	0.99^d^	0.76^c^	0.97^d^	0.98^d^

Colours represent OER ranges: 0-0.54 = ^a^; 50 %, 0.55–0.69 = ^b^; 35 %, 0.70–0.84 = ^c^; 25 %, 0.85–0.99 = ^d^; 15 % 1.0–1.14 = ^e^

## Discussion

Lifestyle diseases, including type 2 diabetes, are without a doubt a growing problem of our times, both locally and globally. The pace of our lives is getting faster – we work harder and longer, which leaves less and less time for leisure. Unfortunately, this lifestyle is not without a cost to our health. As a result, diabetes was the first non-communicable disease in the world to be recognised by the World Health Organisation as an epidemic [12]. This has been confirmed by research findings. The burden of the disease measured in YLLs is higher for diabetes than other non-communicable diseases, such as breast cancer or heart disease. For illustrative purposes, one may consider absolute mortality figures. In 2012, there were 1.5 million deaths worldwide which could be attributed directly to diabetes. It was the eighth leading cause of death for both sexes, and the fifth leading cause of death for women in 2012 [13].

People with uncontrolled diabetes are affected by frequent hypoglycemic episodes, increasing the risk of severe complications in the course of the disease. Looking at the general population of diabetes patients in Poland treated within the healthcare system, the prevalence of severe hypoglycemic episodes is 0.120 per patient per year. In other Central European countries, such episodes are less prevalent – between 0.117 and 0.096 [14]. Moreover, the disease has significant adverse effects on quality of life and social functioning, consequently contributing to the development of disability in a somatic and psychosomatic sense (the so-called vicious cycle of diabetes). This has also been reported in our own studies. Based on our analysis, the number of years lived with disability (YLD) in Poland has been on the rise at a rate of 12 % a year. Global data reveal an average annual growth rate of 8.57 %.

According to the International Diabetes Federation (IDF), over the past decade, the global growth rate of diabetes amounted to 3 % a year. The IDF predicts that by 2035 the population of people with diabetes will rise up to 592 million worldwide. Close to a half, i.e. 46 % of that group, will be yet undiagnosed, and therefore exposed to a high risk of complications as a result of non-treatment. In Europe, there are 56 million people living with diabetes. In recent years, there has been a marked increase in diabetes incidence among children and adolescents [15, 16]. According to the IDF, 8 % of the Polish population suffer from diabetes, with 1/3 of that number remaining undiagnosed [13, 16, 17]. To a large extent, this is related to the increasing prevalence of obesity, in adults and children alike [18]. Studies suggest that the lowest rates of diabetes are observed among people with BMI scores under 25.0. With increasing BMI values, the risk of developing type 2 diabetes goes up, too. Nearly a quarter of adults suffering from diabetes have poor glycemic control, and almost a half are obese (49.1 %) [19]. The rise in BMI values in patients with type 2 diabetes is also associated with a statistically significant increase in the risk of death from cardiovascular causes [20].

According to the WOBASZ study, among people with type 2 diabetes, close to a half are also affected by obesity. In European countries, this problem occurs with a varying degree of severity: in France 49 % of diabetic patients are obese, in Germany – 51 %, in the United Kingdom – 56 %, and in recent years there has been a perceptible rise in these figures. People with diabetes and concomitant obesity also find it harder to control glycated hemoglobin levels. In this patient group, 21 % present HbA1c above 7 %. People with diabetes and concomitant obesity report markedly lower quality of life, having to deal with poorer glycemic control, arterial hypertension, high cholesterol levels and a much greater risk of cardiovascular complications [21].

Since 2012, the cost of diabetes treatment has been on the rise, accounting for approx. 11 % of overall global expenditure on healthcare. In highly developed countries, as much as 75 % of healthcare expenditure dedicated to diabetes goes towards hospital treatment of its complications. In the developing countries, the structure of diabetes-related expenditure is a little different, with a lion’s share of the cost transferred to the patients, who have to pay for their treatment by themselves [22]. This may be the reason for the significant difference between the observed incidence of type 2 diabetes and the expected figures. Projections provide for higher numbers of cases than the actual records. In recent years, this discrepancy has been diminishing, particularly in Poland. This may be due to the growing problem of obesity, as well as the underestimation of financial outlay on treatment. The expenditure on diabetes treatment in Poland, per person, is one of the lowest in Europe. In 2013, it amounted to USD 1037, down from USD 1145 the year before. Also, at present Poland ranks last in Central Europe in terms of the average expenditure per diabetic patient among countries with a similar GDP [23].

Another important and so far unmentioned reason for the growing prevalence of diabetes in Europe is the ageing population [24, 25]. Both highly developed and developing countries will be significantly affected by this phenomenon in the coming decades, hence now is the time to undertake wide-ranging measures aimed at providing capacity to face the consequences of rising healthcare needs in the future.

## Conclusions

Health indicators allow for monitoring changes in the health status of the population. The changes observed in health metrics confirm that the aim of public health and health care measures is not only to extend life, but to extend life in good health. The present analysis made it possible to visualise the number of years of life lost due to premature mortality in the Polish population compared to Central European and Global populations. Type 2 diabetes was demonstrated to impact on shortening lifespans, because the disease has been following a growing trend in the years covered by the analysis compared to other health conditions. As a result, a decreasing proportion of the population, irrespective of geographic location, is able to enjoy good health, free from limitations and disability. Looking at the data from the *Global Burden of Disease* study, the differences between observed and expected YLL, YLD, DALY figures for type 2 diabetes have been diminishing in recent years. Hence, the projections presented by the GBD may be treated as a reliable source and foundation for devising health policies aimed at reducing the numbers of diabetes patients as well as the complications, comorbidities and deaths as a consequence of that disease.

## Recommendations

Based on their investigations and analysis of literature, the authors recommend [12, 13, 22, 23]:

- promoting knowledge on the risk factors for diabetes and on the disease itself in society, coupled with screening for diabetes in risk groups within the framework of general practice and occupational medicine services;
- developing education and counselling in diabetes care and giving high priority to patient education tailored to the different age groups of diabetic patients;
- increasing the availability of diabetes medications eligible for refund, including but not limited to novel anti-diabetes agents reducing the risk of cardiovascular complications and hypoglycemia, as well as drugs improving quality of life for patients receiving insulin treatment;
- ensuring continued public funding of treatment with the use of personal insulin pumps, and improved financing of insulin pump consumables, financing of continuous glucose monitoring systems.

## Data Availability

Data from Global Burden of Disease.

## Abbreviations

DM: Diabetes mellitus
DALY: Disability-adjusted life years
YLD: Years lived with disability
YLL: Years of life lost
GBD: Global Burden of Disease
HDI: Human development index
UI: Uncertainty interval
